# Supplementary material for: A Holistic Strategy of Mother and Child Health Care to Improve the Coverage of Routine and Polio Immunization in Pakistan: Results from a Demonstration Project
Source: Vaccines (Basel). 2024 Jan 16;12(1):89. doi: 10.3390/vaccines12010089 (PMC10819799; doi:10.3390/vaccines12010089)
Supplement: Supplementary file 1 [file vaccines-12-00089-s001.zip › vaccines-2724929-supplementary.pdf]

Table S1. Baseline immunization status by gender for children younger than 5 years and their families (N = 122,950).

Immunization Status	Gender	Overall	Karachi	KP	Baluchistan
Full	Boys	28,109 (41.7%)	11,021 (57.2%)	16,860 (35.6%)	228 (26.7%)
	Girls	23,094 (41.6%)	9988 (54.6%)	12,890 (35.5%)	216 (26.4%)
Partial	Boys	23,642 (35.5%)	5496 (28.5%)	18,135 (38.3%)	311 (35.0%)
	Girls	19,666 (35.5%)	5418 (29.6%)	13,962 (38.4%)	286 (34.9%)
No routine EPI (Zero dose children)	Boys	15,429 (22.9%)	2766 (14.3%)	12,313 (26.1%)	350 (39.3%)
	Girls	12,710 (22.9%)	2886 (15.8%)	9507 (26.2%)	317 (38.7%)

Abbreviations: KP = Khyber Pakhtunkhwa.

Table S2. Vaccine status of children younger than 5 years at Baseline (N = 122, 950) and Endline (N = 133,996).

**Province**	**Karachi**		**KP**		**Baluchistan**	
**Survey**	**Baseline (BL)**	**Endline (EL)**	**Baseline (BL)**	**Endline (EL)**	**Baseline (BL)**	**Endline (EL)**
Children <5 years old	37,575	34,912	83,667	97,712	1708	1372
**Immunization factor**						
Full immunization	21,009 (55.9%)	23,610 (67.6%)	29,750 (35.6%)	58,995 (60.4%)	444 (26.0%)	829 (60.4%)
BCG	31,563 (84.0%)	31,656 (90.7%)	38,263 (45.7%)	71,145 (72.8%)	718 (42.0%)	1142 (83.2%)
**OPV**						
0	31,260 (83.2%)	31,393 (89.9%)	48,735 (58.3%)	72,555 (74.3%)	978 (57.3%)	1131 (82.4%)
1	28,757 (76.5%)	29,469 (84.4%)	50,467 (60.3%)	76,467 (78.3%)	966 (56.6%)	1133 (82.6%)
2	26,809 (71.4%)	27,914 (80.0%)	49,254 (58.9%)	72,175 (73.9%)	903 (52.9%)	1063 (77.5%)
3	25,123 (66.9%)	26,671 (76.4%)	48,302 (57.7%)	70,468 (72.1%)	803 (47.0%)	982 (71.6%)
**Pentavalent**						
1	28,987 (77.0%)	29,616 (84.8%)	35,533 (42.5%)	72,352 (74.1%)	716 (41.9%)	1131 (82.4%)
2	26,941 (71.7%)	28,037 (80.3%)	34,029 (40.7%)	67,442 (69.0%)	617 (36.1%)	1068 (77.8%)
3	25,258 (67.2%)	26,789 (76.7%)	33,078 (39.5%)	64,620 (66.1%)	540 (31.6%)	985 (71.8%)
**PCV**						
1	21,293 (56.7%)	24,765 (70.9%)	35,023 (41.9%)	72,095 (73.8%)	693 (40.6%)	1134 (82.7%)
2	19,706 (52.4%)	23,438 (67.1%)	33,651 (40.2%)	67,191 (68.8%)	613 (35.9%)	1065 (77.6%)

3	18,399 (49.0%)	22,398 (64.2%)	32,768 (39.2%)	64,580 (66.1%)	534 (31.3%)	985 (71.8%)
**Measles**						
1	21,372 (56.9%)	23,788 (68.1%)	44,838 (53.6%)	72,608 (74.3%)	519 (30.4%)	923 (67.3%)
2	17,135 (45.6%)	20,008 (57.3%)	36,438 (43.6%)	57,985 (59.3%)	438 (25.6%)	722 (52.6%)
IPV	6702 (17.8%)	17,656 (50.6%)	4422 (5.3%)	36,099 (36.9%)	587 (34.4%)	1013 (73.8%)

Abbreviations: Bacillus Calmette-Guerin vaccine, IPV = Inactivated Polio vaccine, KP = Khyber Pakhtunkhwa, OPV = Oral Polio vaccine, PCV = Pneumococcal conjugate vaccine.

Table S3. Characteristics of health camp attendees and vaccines delivered by region.

Characteristic	Karachi (N = 681,817)	KP (N = 181,244)	Baluchistan (N = 72,552)	*p*-Value
Female	439,634 (64.5%)	104,531 (57.7%)	50,167 (69.2%)	<0.001 [*,†,‡]
Children <5 years old	321, 641 (47.2%)	125,146 (69.1%)	33,975 (46.8%)	<0.001 [*,‡]
Primary source of info: community mobilization	558,809 (82.0%)	133,179 (73.5%)	56,989 (78.6%)	<0.001 [*,†,‡]
Received antenatal care (ANC)	6409 (0.9%)	9894 (5.5%)	236 (0.3%)	<0.001 [*,†,‡]
Vaccination card available	86,979 (12.8%)	33,327 (18.4%)	15,638 (21.6%)	<0.001 [*,†,‡]
**Vaccinations delivered**				
BCG	12,074 (1.8%)	20,561 (11.3%)	4015 (5.5%)	<0.001 [*,†,‡]
**OPV**				
0	7887 (1.2%)	9333 (5.2%)	1078 (1.5%)	<0.001 [*,†,‡]
1	8525 (1.3%)	26,957 (14.9)	1196 (1.7%)	<0.001 [*,†,‡]
2	5027 (0.7%)	8118 (4.5%)	910 (1.3%)	<0.001 [*,†,‡]
3	62,621 (9.2%)	12,925 (7.1%)	2707 (3.7%)	<0.001 [*,†,‡]
**Pentavalent**				
1	9690 (1.4%)	24,165 (13.3%)	2639 (3.6%)	<0.001 [*,†,‡]
2	4642 (0.7%)	7490 (4.1%)	1182 (1.6%)	<0.001 [*,†,‡]
3	11,337 (1.7%)	8674 (4.8%)	3020 (4.2%)	<0.001 [*,†,‡]
**PCV**				
1	9209 (1.4%)	22,873 (12.6%)	2609 (3.6%)	<0.001 [*,†,‡]
2	4528 (0.7%)	7417 (4.1%)	1182 (1.6%)	<0.001 [*,†,‡]
3	11,130 (1.6%)	7423 (4.1%)	3016 (4.2%)	<0.001 [*,†]
**Measles**				
1	26,531 (3.9%)	30,008 (16.6%)	3751 (5.2%)	<0.001 [*,†,‡]
2	53, 289 (7.8%)	9273 (5.1%)	16,408 (22.6%)	<0.001 [*,†,‡]
IPV	128,812 (18.9%)	83,160 (45.9%)	8466 (11.7%)	<0.001 [*,†,‡]
**Vaccination refusals**				
Routine immunization	6655 (1.0%)	8 (<0.1%)	110 (0.2%)	<0.001 [*,†,‡]
OPV	463 (0.1%)	6 (<0.1%)	88 (0.1%)	<0.001 [*,†,‡]
IPV	947 (0.1%)	25,484 (14%)	48 (0.1%)	<0.001 [*,†,‡]

Abbreviations: Bacillus Calmette-Guerin vaccine, IPV = Inactivated Polio vaccine, KP = Khyber Pakhtunkhwa, OPV = Oral Polio vaccine, PCV = Pneumococcal conjugate vaccine; * $p < 0.001$ (Bonferroni corrected) for Karachi vs. KP; † $p < 0.001$ (Bonferroni corrected) for Karachi vs. Baluchistan; ‡ $p < 0.001$ (Bonferroni corrected) for KP vs. Baluchistan.

Table S4. Crude estimates of routine immunization coverage at Baseline (BL) and Endline (EL) for children under five years of age overall and by region.

	Overall				Karachi				KP				Baluchistan			
**Immunization Factor**	**BL (95% CI)**	**EL (95% CI)**	**EL−BL (95% CI)**	***p***	**BL (95% CI)**	**EL (95% CI)**	**EL−BL (95% CI)**	***p***	**BL (95% CI)**	**EL (95% CI)**	**EL−BL (95% CI)**	***p***	**BL (95% CI)**	**EL (95% CI)**	**EL−BL (95% CI)**	***p***
Full immunization	41.6 (41.4; 41.9)	62.3 (62; 62.5)	20.6 (20.2; 21)	<0.001	55.9 (55.4; 56.4)	67.6 (67.1; 68.1)	11.7 (11; 12.4)	<0.001	35.6 (35.2; 35.9)	60.4 (60.1; 60.7)	24.8 (24.4; 25.3)	<0.001	26 (24; 28.1)	60.4 (57.8; 63)	34.4 (31.1; 37.7)	<0.001
BCG	57.4 (57.1; 57.7)	77.6 (77.4; 77.8)	20.2 (19.8; 20.5)	<0.001	84.0 (83.6; 84.4)	90.7 (90.4; 91.0)	6.7 (6.2; 7.2)	<0.001	45.7 (45.4; 46.1)	72.8 (72.5; 73.1)	27.1 (26.6; 27.5)	<0.001	42 (39.7; 44.4)	83.2 (81.2; 85.1)	41.2 (38.1; 44.3)	<0.001
**OPV**																
0	65.9 (65.6; 66.1)	78.4 (78.2; 78.6)	12.6 (12.2; 12.9)	<0.001	83.2 (82.8; 83.6)	89.9 (89.6; 90.2)	6.7 (6.2; 7.2)	<0.001	58.2 (57.9; 58.6)	74.3 (74; 74.5)	16 (15.6; 16.4)	<0.001	57.3 (54.9; 59.6)	82.4 (80.3; 84.4)	25.2 (22.1; 28.3)	<0.001
1	65.9 (65.6; 66.1)	80.4 (80.1; 80.6)	14.5 (14.2; 14.8)	<0.001	77.9 (77.4; 78.3)	85.6 (85.2; 85.9)	7.7 (7.1; 8.3)	<0.001	60.7 (60.4; 61)	78.5 (78.2; 78.7)	17.8 (17.3; 18.2)	<0.001	57.5 (55.1; 59.8)	84.3 (82.3; 86.2)	26.9 (23.8; 30)	<0.001
2	63.8 (63.6; 64.1)	76.5 (76.2; 76.7)	12.6 (12.3; 13)	<0.001	73.8 (73.3; 74.2)	82.4 (82.0; 82.8)	8.6 (8; 9.2)	<0.001	59.7 (59.3; 60)	74.4 (74.1; 74.6)	14.7 (14.3; 15.1)	<0.001	54.5 (52.1; 56.9)	80.6 (78.4; 82.7)	26.1 (22.9; 29.3)	<0.001
3	61.9 (61.6; 62.2)	74.4 (74.1; 74.6)	12.5 (12.1; 12.8)	<0.001	69.9 (69.4; 70.4)	79.3 (78.8; 79.7)	9.3 (8.7; 10)	<0.001	58.7 (58.4; 59)	72.7 (72.4; 73)	14 (13.5; 14.4)	<0.001	48.4 (46; 50.8)	74.9 (72.5; 77.2)	26.5 (23.1; 29.9)	<0.001
**Pentavalent**																
1	53.6 (53.3; 53.8)	77.4 (77.1; 77.6)	23.8 (23.5; 24.2)	<0.001	78.5 (78.1; 78.9)	86.0 (85.6; 86.4)	7.5 (7; 8.1)	<0.001	42.8 (42.4; 43.1)	74.2 (74; 74.5)	31.5 (31; 31.9)	<0.001	42.7 (40.4; 45.1)	84.2 (82.1; 86)	41.5 (38.4; 44.5)	<0.001
2	51.1 (50.8; 51.4)	73 (72.8; 73.2)	21.9 (21.5; 22.3)	<0.001	74.2 (73.7; 74.6)	82.8 (82.4; 83.2)	8.6 (8; 9.2)	<0.001	41.3 (40.9; 41.6)	69.5 (69.2; 69.8)	28.2 (27.8; 28.7)	<0.001	37.5 (35.2; 39.9)	80.9 (78.6; 82.9)	43.4 (40.2; 46.5)	<0.001
3	49.1 (48.8; 49.4)	70 (69.8; 70.3)	20.9 (20.5; 21.3)	<0.001	70.3 (69.8; 70.8)	79.6 (79.2; 80.1)	9.3 (8.7; 10)	<0.001	40.3 (39.9; 40.6)	66.6 (66.3; 66.9)	26.4 (25.9; 26.8)	<0.001	32.8 (30.6; 35.1)	75.1 (72.6; 77.3)	42.2 (39; 45.5)	<0.001
**PCV**																
1	46.8 (46.5; 47.1)	73.5 (73.3; 73.8)	26.7 (26.4; 27.1)	<0.001	57.5 (57.0; 58.0)	71.8 (71.3; 72.3)	14.2 (13.5; 14.9)	<0.001	42.2 (41.8; 42.5)	74 (73.7; 74.3)	31.8 (31.4; 32.3)	<0.001	41.4 (39; 43.7)	84.4 (82.4; 86.3)	43.1 (40; 46.1)	<0.001
2	44.8 (44.5; 45)	69.3 (69.1; 69.6)	24.6 (24.2; 24.9)	<0.001	54.1 (53.6; 54.6)	69.0 (68.5; 69.5)	14.9 (14.2; 15.7)	<0.001	40.8 (40.5; 41.2)	69.3 (69; 69.5)	28.4 (28; 28.9)	<0.001	37.2 (34.9; 39.5)	80.7 (78.5; 82.7)	43.5 (40.4; 46.7)	<0.001
3	43.1 (42.8; 43.4)	66.6 (66.4; 66.9)	23.5 (23.2; 23.9)	<0.001	51.1 (50.5; 51.6)	66.4 (65.9; 66.9)	15.4 (14.6; 16.1)	<0.001	39.9 (39.5; 40.2)	66.6 (66.3; 66.9)	26.7 (26.3; 27.2)	<0.001	32.4 (30.2; 34.7)	75.1 (72.6; 77.3)	42.7 (39.4; 45.9)	<0.001
**Measles**																
1	59.3 (59; 59.6)	77.6 (77.4; 77.9)	18.3 (18; 18.7)	<0.001	65.5 (65.0; 66.0)	76.9 (76.4; 77.4)	11.4 (10.7; 12.1)	<0.001	57.2 (56.8; 57.5)	77.9 (77.6; 78.1)	20.7 (20.3; 21.1)	<0.001	34.7 (32.3; 37.2)	78 (75.5; 80.3)	43.3 (39.9; 46.7)	<0.001
2	52.9 (52.6; 53.3)	69.8 (69.5; 70)	16.8 (16.4; 17.2)	<0.001	59.1 (58.5; 59.7)	73.0 (72.4; 73.5)	13.9 (13.1; 14.6)	<0.001	57.2 (56.8; 57.5)	68.6 (68.3; 68.9)	17.8 (17.3; 18.3)	<0.001	33.2 (30.6; 35.8)	74.2 (71.3; 76.9)	41 (37.2; 44.8)	<0.001
IPV	10.4 (10.2; 10.6)	46.2 (45.9; 46.4)	35.8 (35.4; 36.1)	<0.001	24.4 (23.8; 24.9)	58.5 (57.9; 59.0)	34.1 (33.4; 34.9)	<0.001	5.3 (5.1; 5.4)	41.5 (41.1; 41.8)	36.2 (35.8; 36.5)	<0.001	34.4 (32.2; 36.7)	73.8 (71.4; 76.1)	39.5 (36.2; 42.7)	<0.001

Abbreviations: Bacillus Calmette-Guerin vaccine, CI = Confidence interval, IPV = Inactivated Polio vaccine, KP = Khyber Pakhtunkhwa, OPV = Oral Polio vaccine, PCV = Pneumococcal conjugate vaccine.
